# Stromal Derived Factor-1/CXCR4 Axis Involved in Bone Marrow Mesenchymal Stem Cells Recruitment to Injured Liver

**DOI:** 10.1155/2016/8906945

**Published:** 2016-01-12

**Authors:** Kuai Xiao Ling, Li Peng, Zhang Jian Feng, Cao Wei, Yuan Wei Yan, Shao Nan, Guan Cheng Qi, Wang Zhi Wei

**Affiliations:** ^1^Department of Gastroenterology, Nantong University Affiliated Hospital, Nantong, Jiangsu 226001, China; ^2^Department of General Surgery, Nantong University Affiliated Hospital, Nantong, Jiangsu 226001, China

## Abstract

The molecular mechanism of bone marrow mesenchymal stromal stem cells (BMSCs) mobilization and migration to the liver was poorly understood. Stromal cell-derived factor-1 (SDF-1) participates in BMSCs homing and migration into injury organs. We try to investigate the role of SDF-1 signaling in BMSCs migration towards injured liver. The expression of CXCR4 in BMSCs at mRNA level and protein level was confirmed by RT-PCR, flow cytometry, and immunocytochemistry. The SDF-1 or liver lysates induced BMSCs migration was detected by transwell inserts. CXCR4 antagonist, AMD3100, and anti-CXCR4 antibody were used to inhibit the migration. The Sprague-Dawley rat liver injury model was established by intraperitoneal injection of thioacetamide. The concentration of SDF-1 increased as modeling time extended, which was determined by ELISA method. The Dir-labeled BMSCs were injected into the liver of the rats through portal vein. The cell migration in the liver was tracked by *in vivo* imaging system and the fluorescent intensity was measured. *In vivo*, BMSCs migrated into injured liver which was partially blocked by AMD3100 or anti-CXCR4 antibody. Taken together, the results demonstrated that the migration of BMSCs was regulated by SDF-1/CXCR4 signaling which involved in BMSCs recruitment to injured liver.

## 1. Introduction

Bone marrow mesenchymal stem cells (BMSCs) are nonhematopoietic progenitor cells, capable of differentiating into bone, adipose, and cartilage [1]. They also support the survival and proliferation of hematopoietic stem cells [2]. Furthermore, studies indicated that BMSCs were able to recruit to the site of injury and contribute to the repair process such as bone, heart, lung, and liver [3–6]. BMSCs are currently being investigated in numerous clinical trials of tissue repair and various immunological disorders based on their ability to secrete trophic factors and to modulate inflammatory responses. BMSCs migration and recruitment are crucial to the success of BMSCs-based therapies. Migratory mechanisms need to be elucidated before BMSCs can be exploited therapeutically.

Chemokines are the most important factors controlling cellular migration. Stromal derived factor-1 (SDF-1, also called CXCL-12), acting via its receptor CXCR4 play an important role in BMSCs homing to bone marrow. Mobilization of BMSCs from bone marrow to peripheral blood, and thence to injured tissues, may be down an SDF-1 concentration gradient [7, 8]. A number of studies have shown that SDF-1 is critical for BMSCs homing in injured tissue through interaction with its receptor CXCR4 [9–12].

It was reported that the injured liver releases chemokines, such as stroma-derived factor-1 (SDF-1), hepatocellular growth factor (HGF), and others, to participate in the concert of extrahepatic cells homing to the liver [13, 14]. It was shown that levels of SDF-1 in injured liver increased, which attracting CD133^+^ BMSC, that are positive for the SDF-1 receptor CXCR4 [15].

In this study, we hypothesized that SDF-1 of injured liver promotes BMSCs migration towards the liver via its receptor CXCR4. We explored the expression of CXCR4 in BMSCs.* In vitro* SDF-1 induced BMSCs migration was investigated and* in vivo* BMSCs migration towards injured liver was also tested. Our results demonstrated the role of SDF-1/CXCR4 axis in BMSCs migration towards injured liver.

## 2. Material and Methods

### 2.1. Cell Lines and Cell Culture

BMSCs were from Cyagen Biosciences Inc. (Santa Clara, CA, USA; http://www.cyagen.com/) and maintained in alpha minimal essential medium (aMEM; Gibco, Invitrogen, Rockville, MD; http://www.lifetechnologies.com/), supplemented with 10% fetal bovine serum (FBS), 100 U/mL penicillin, 100 mg/mL streptomycin, and 2 mM L-glutamine (Gibco, Invitrogen) as described previously [16]. 293 T cells were from ATCC (Beijing, China; http://www.atcc.org/) and cultured in Dulbecco's Modified Eagle's Medium (DMEM; Gibco) with 10% FBS, 2 mM L-glutamine, and 1% penicillin/streptomycin.

### 2.2. Reverse Transcription PCR

Total cellular RNA was isolated using a RNeasy Mini Kit (Qiagen, Valencia, CA; https://www.qiagen.com/) and treated with a DNA-free kit (Ambion, Austin, TX; http://www.lifetechnologies.com/) to remove potential contamination of genomic DNA. A total of 500 ng of RNA was used as a template for reverse transcription with Reverse Transcription System (Promega, Madison, WI; http://www.promega.com/). 100 ng of cDNA was used for a standard PCR reaction. A housekeeping gene, glyceraldehyde-3-phosphate dehydrogenase (GAPDH), was used as a control for the PCR efficiency of each sample. The PCR step was performed using PCR Master Mix kit (Promega, Madison, WI), and the PCR products were detected and analyzed by 2% agarose gel electrophoresis. 293 T cells were as negative control.

CXCR4 primers were as follows: forward 5′-ATG GAG GGG ATC AGT ATA TAC AC-3′ and reverse 5′-TGG AGT GTG CTA TGT TGG CGT CT-3′ (product 668 bp); GAPDH primers were forward 5′-ACC-ACA-GTC-CAT-GCC-ATC-AC-3′ and reverse 5′-TCC-ACC-ACC-CTG-TTG-CTG-TA-3′ (product 450 bp).

### 2.3. Flow Cytometry

BMSCs were fixed with 4% paraformaldehyde (Sigma-Aldrich, Saint Louis, MO, USA; http://www.sigmaaldrich.com/), permeabilized with 0.5% Triton X-100 (Sigma-Aldrich), and stained with mouse monoclonal anti-human CXCR4 antibody (R&D Systems); at this step PBS and isotype antibody (R&D Systems) were used as negative control and then followed by anti-mouse IgG (FITC; Sigma-Aldrich) according to the manufacturer's instructions. Cells were analyzed on a FACSCalibur with CellQuest software (BD Biosciences).

### 2.4. Immunocytochemistry

Cells were cultured in chamber slides and then were fixed in 4% paraformaldehyde in PBS for 15 minutes, permeabilized with 0.1% Triton X-100 for 10 minutes, and then blocked for 1 hour at room temperature in PBS containing 5% goat serum (Invitrogen, Rockville, MD). Samples were then incubated in blocking buffer containing mouse monoclonal anti-human CXCR4 antibody or isotype antibody (R&D Systems) for 2 hours at room temperature and washed three times with PBS for 15 minutes. Cells were then incubated with secondary anti-mouse antibody conjugated with FITC (1 : 1000, Molecular Probes, Eugene, OR; http://www.lifetechnologies.com/) for 1 hour at room temperature. The samples were washed as above and mounted with 6-diamidino-2-phenylindole (DAPI; DAKO, Carpinteria, CA; http://www.dako.com/) containing mounting solution. The cells were examined under a Nikon Eclipse E600 fluorescence microscope (Nikon, Tokyo, Japan; http://www.nikon.com/).

### 2.5. Chemotaxis Assays

BD FluoroBlok inserts (BD Falcon Labware, Franklin Lakes, NJ, http://www.bdbiosciences.com/) contain fluorescence blocking PET track-etched membranes with 8.0 *μ*m pores. This invasion system allowed for real-time viewing of cell invasion without the need to end the experiment and process the membranes. The FluoroBlok membrane prevents the transmission of light to cells on top of the membrane; thus, only invaded fluorescent-expressing cells can be viewed. Cells were serum-starved overnight before being harvested for invasion assays. BMSCs were labeled with PKH26GL dye (Sigma-Aldrich) and then were plated on top of the chamber layer at a concentration of 4 × 10^5^ cells/mL. In the bottom chamber, SDF-1 (R&D Systems Minneapolis, MN; http://www.rndsystems.com/) or liver lysates (taken at the 12th week after TAA injection) were used as chemoattractant. For neutralization studies, cells were incubated with anti-CXCR4 monoclonal antibody (R&D Systems) at 20 *μ*g/mL, 40 *μ*g/mL, 80 *μ*g/mL, and 96 *μ*g/mL or AMD3100 (Plerixafor) at 24 *μ*g/mL, 48 *μ*g/mL, and 96 *μ*g/mL (Sigma-Aldrich). AMD3100 is an antagonist of CXCR4, which disrupted binding of SDF-1 to CXCR4 by competing binding site, thus blocking the physiological function of SDF-1/CXCR4 axis. Six duplicated wells were in each experiment. Invaded cells with red fluorescence were viewed at 48 hours after chemoattractant addition under a Nikon Eclipse E600 fluorescence microscope (Nikon, Tokyo, Japan; http://www.nikon.com/). Cells were counted from entire membranes at 40x. The migrated cell number was expressed as mean ± SD.

### 2.6. Animal Studies

#### 2.6.1. Animals

Six- to eight-week-old, between 160 and 200 g, wide-type female Sprague-Dawley (SD) rats were obtained from the Animal Research Center of Fudan University in China. They were randomized and divided into different groups, six rats in each group. Rats were maintained in the animal care facility of our institution. All experiments were approved by the Animal Experimentation Ethics Committee (AEEC) of Nantong University Affiliated Hospital.

#### 2.6.2. Liver Injury Model

Liver injury model was induced in rats by intraperitoneal (IP) injections with sterile solutions of thioacetamide (TAA) (Sigma-Aldrich), which was dissolved in 0.9% saline, administered twice weekly. During the initial week, rats were IP injected with TAA 0.25 g/kg body weight biweekly. During the following 11 weeks, rats were IP injected with TAA 0.20 g/kg body weight biweekly [17]. The serum Alanine aminotransferase (ALT) and aspartate aminotransferase (AST) levels of rats after TAA injection were detected by automatic biochemistry analyzer (Beckman Coulter) to confirm liver injury at 0 weeks, 4 weeks, 8 weeks, and 12 weeks. And the histological sections of livers were stained with hematoxylin-eosin staining (HE staining) to observe pathological changes of the livers.

#### 2.6.3. BMSCs Injection

Rats were placed under anesthesia using isoflurane through inhalation. Before cell injection, BMSCs were labeled with 1,1-dioctadecyl-3,3,3,3-tetramethylindotricarbocyanine iodide (DiR) dye (Invitrogen). Dir-labeled BMSCs before injection were as positive control and rats without cell injection were as negative control. First, the ways of cells injection were compared between portal vein injection (PVI) and tail vein injection (TVI). More migrated cells were found in liver through PVI, so PVI was selected in the following cell migration study. Six rats were in each group. Each rat was injected with 1 × 10^6^ BMSCs through portal vein using a 16 G syringe. The cells migration in normal and injured liver was also compared. For neutralization studies, cells were incubated with anti-CXCR4 monoclonal antibody (R&D Systems) before injection or with AMD3100 during injection. At the end, a laparotomy was performed and the livers were dissected.

#### 2.6.4. *In Vivo* Distribution of the Transplanted Cells

The location and the fluorescent strength of the transplanted cells labeled with dye DiR (excitation/emission: 748/780 nm) were detected by the Kodak* In-Vivo* Multispectral Imaging System FX (excitation filter/emission filter: ex730/em750WA) at 24 h after cell injection. First, the fluorescent intensity of whole rats was checked. Then, the rats were executed and the livers were perfused with saline (PBS) to remove contaminating blood which might contain unmigrated BMSCs. And then the fluorescent intensity of dissected liver was measured. Dir-labeled BMSCs before injection were as positive control and rats without cell injection were as negative control. At the end of each acquisition a photographic image was obtained. The data were analyzed with Photovision software, which superimposes the signal on the photographic image. The most intense fluorescent signal detected is shown in red, whereas the weakest signal is shown in blue.

### 2.7. Enzyme-Linked Immunosorbent Assay for SDF-1 Level of Liver Lysates

Liver extracts at 0 weeks, 4 weeks, 8 weeks, and 12 weeks after TAA injection were prepared by cell lysis with 25 mM Tris (pH 7.5), 1% Triton X-100, 0.5 mM EDTA, 150 mM NaCl, 10 mM NaF, and protease inhibitor cocktail (1% PMSF; Sigma-Aldrich). Total proteins in these extracts were quantified by Bradford assay and equal protein amounts were assayed for SDF-1 level with SDF-1 enzyme-linked immunosorbent assay (ELISA) kit (R&D Systems) according to the manufacturer's instructions.

### 2.8. Statistics

Statistical analysis and graphing were performed using SPSS 12. All results are expressed as mean ± SEM. Statistical significance was determined by Student's *t*-test; Significance was defined as *P* < 0.05.

## 3. Results

### 3.1. Expression of CXCR4 in BMSCs

**Figure 1:**
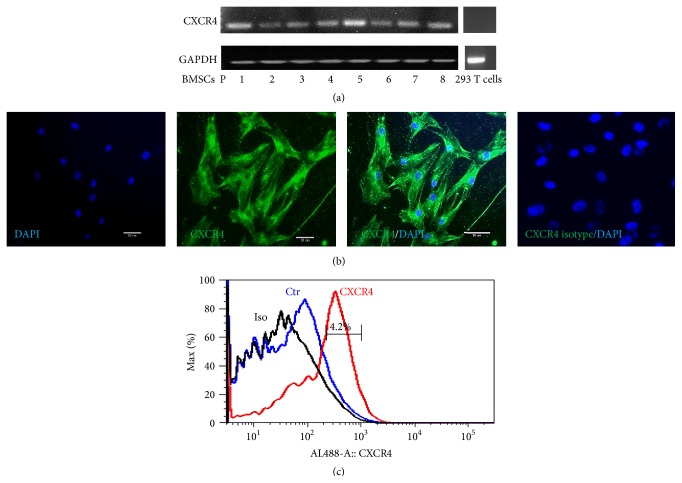
Expression of CXCR4 in BMSCs. (a) RT-PCR results of CXCR4 expression in BMSCs from passages 1 to 8; CXCR4 was stably expressed; 293T cells as negative control; (b) immunocytochemistry results of CXCR4 expression, which indicated both membrane and intracellular CXCR4 expression; (c) the result of flow cytometry indicated only 4.2% of cells were positive for membrane CXCR4.

The expression of CXCR4 in BMSCs was examined at mRNA and protein level from passages 1 to 8 with RT-PCR, immunocytochemistry, and flow cytometry. CXCR4 was stably expressed in BMSCs at mRNA level from passages 1 to 8; 293 T cells were as negative control (Figure 1(a)). Before staining with CXCR4 antibody, BMSCs were permeabilized with 0.1% Triton X-100, so the immunocytochemistry results indicated all the CXCR4 expression, on the membrane and intracellularly. CXCR4 were found in 100% of cells (Figure 1(b)). Only membrane CXCR4 positive BMSCs could be detected by flow cytometry. The result of flow cytometry indicated 4.2% of cells were membrane CXCR4 positive (Figure 1(c)).

### 3.2. SDF-1 Induce BMSCs Migration* In Vitro*

**Figure 2:**
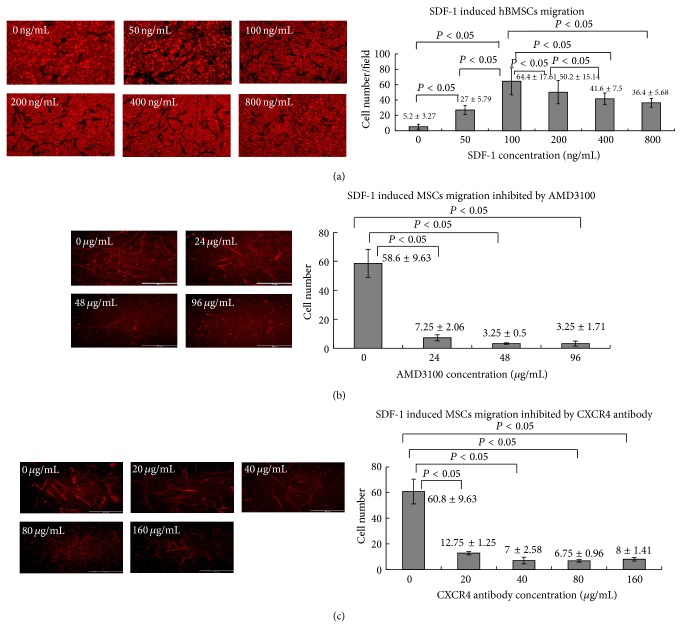
SDF-1 induces BMSCs migration* in vitro*. (a) SDF-1 at different concentration; the migrated cells were observed under fluorescence microscope and the graph indicated the number of migrated cells; (b) SDF-1 at 100 ng/mL; AMD3100 was added at different concentration, the migrated cells were observed and counted under fluorescence microscope and the graph indicated the number of migrated cells; (c) SDF-1 at 100 ng/mL; anti-CXCR4 antibody was added at different concentration, the migrated cells were observed and counted.

In cell transwell system, SDF-1 added in bottom chamber was at 0 ng/mL, 50 ng/mL, 100 ng/mL, 200 ng/mL, 400 ng/mL, and 800 ng/mL; the number of migrated cells were counted under fluorescence microscopes, which were 5.2 ± 3.27, 27 ± 5.79, 64.4 ± 17.61, 50.2 ± 15.14, 41.6 ± 7.5, and 36.4 ± 5.68, respectively. The concentration of SDF-1 lower than 100 ng/mL, the number of migrated cells increased as the concentration increased, and the differences were statistically significant (all *P* < 0.05). However, after the concentration was above 100 ng/mL the number of migrated cells decreased because too much SDF-1 would complete the binding site (Figure 2(a)).

To demonstrate that the migration was an SDF-1 dependent effect, CXCR4 antagonist AMD3100 or anti-CXCR4 antibody was added. The concentration of SDF-1 at 100 ng/mL, after adding AMD3100 at 24 *μ*g/mL, 48 *μ*g/mL, and 96 *μ*g/mL, the number of migrated cells decreased from 58.6 ± 9.63 to 7.25 ± 2.06, 3.25 ± 0.5, and 3.25 ± 1.71, and the differences were statistically significant (all *P* < 0.05) (Figure 2(b)). After adding anti-CXCR4 antibody at 20 *μ*g/mL, 40 *μ*g/mL 80 *μ*g/mL, and 96 *μ*g/mL, the number of migrated cells decreased from 60.8 ± 9.63 to 12.75 ± 1.25, 7 ± 2.58, 6.75 ± 0.96, and 8 ± 1.41, respectively. The differences were statistically significant (all *P* < 0.05) (Figure 2(c)). These results indicated that SDF-1/CXCR4 was involved in the hBMSCs migration.

### 3.3. Liver Injury after TAA Injection and SDF-1 Levels of Rats Liver Lysates

**Figure 3:**
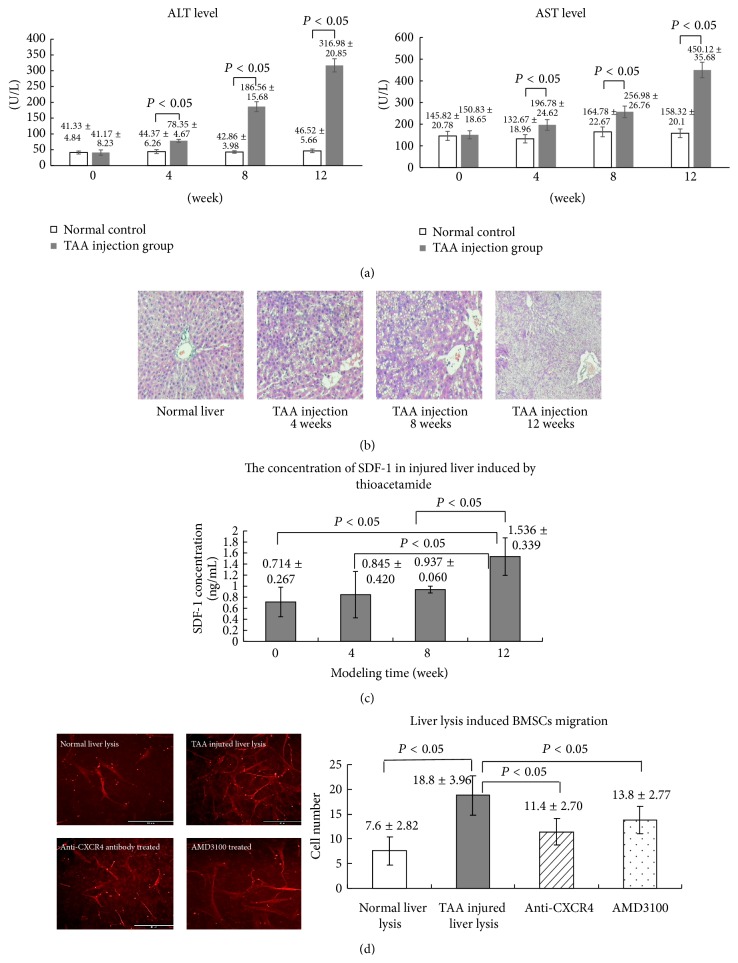
Injured liver lysates induce BMSCs migration* in vitro*. (a) Changes of serum ALT and AST level after TAA injection; (b) HE stained liver tissue sections after TAA injection; (c) the concentrations of SDF-1 of liver lysates detected by ELISA at different time point of TAA induced liver injury; (d) the migrated cells induced by normal liver lysates and TAA injured liver lysates. More cell migration was induced by TAA injured liver lysates which could be partially blocked by AMD3100 (24 *μ*g/mL) or anti-CXCR4 antibody (20 *μ*g/mL). The migrated cells were observed under fluorescence microscope and the graph indicated the number of migrated cells.

Liver injury model was induced in rats by IP injections of TAA. After TAA injection the serum level of ALT at 0 weeks, 4 weeks, 8 weeks, and 12 weeks was 41.17 ± 8.23 U/L, 78.35 ± 4.67 U/L, 186.56 ± 15.68 U/L, and 316.98 ± 20.85 U/L, respectively; and that of the controls was 41.33 ± 4.84 U/L, 44.37 ± 6.62 U/L, 42.86 ± 3.98 U/L, and 46.52 ± 5.66 U/L, respectively. The differences in ALT level between TAA injection group and control group at 4 weeks, 8 weeks, and 12 weeks were statistically significant (*P* < 0.05) (Figure 3(a)). The serum level of AST was 150.83 ± 18.65 U/L, 196.78 ± 24.62 U/L, 256.98 ± 26.76 U/L, and 450.12 ± 35.68 U/L at 0 weeks, 4 weeks, 8 weeks, and 12 weeks after TAA injection, respectively; and that of the controls was 145.82 ± 20.78 U/L, 132.67 ± 18.96 U/L, 164.78 ± 22.67 U/L, and 168.32 ± 20.1 U/L, respectively. The differences between two groups at 4 weeks, 8 weeks, and 12 weeks were statistically significant (*P* < 0.05) (Figure 3(a)). After TAA injection, the serum level of AST and ALT significantly increased (Figure 3(a)), which indicated liver injury. The livers of rats were collected at 0, 4, 8, and 12 weeks after TAA injection. The pathological results also indicated liver injury; there were vacuole degeneration (at 8 weeks), microvascular disintegration (at 8 weeks), tissue necrosis, and disruption of general architecture (at 12 weeks) (Figure 3(b)). The SDF-1 concentrations of livers lysates were determined by ELISA. The SDF-1 concentrations of livers lysates at 0, 4, 8, and 12 weeks were 0.714 ± 0.267 ng/mL, 0.845 ± 0.420 ng/mL, 0.937 ± 0.060 ng/mL, and 1.536 ± 0.339 ng/mL (Figure 3(c)). These results indicated that SDF-1 level increased in TAA induced liver injury.

### 3.4. BMSCs Migration Induced by Liver Extracts* In Vitro*

To explore whether SDF-1 of liver lysates is involved in recruitment of BMSCs, a migration experiment was performed* in vitro* with BMSCs and liver lysates from rats treated by TAA for 12 weeks. The number of migrated cells caused by liver lysates from untreated normal rats was 7.6 ± 2.82 and that by TAA induced injured liver lysates was 18.8 ± 3.96; the difference was statistically significant (*P* < 0.05) (Figure 3(d)). As we demonstrated, SDF-1 level of liver lysates from rats treated by TAA increased, so the question was whether the increased cell number was caused by SDF-1. CXCR4 antagonist AMD3100 or anti-CXCR4 antibody was added in the medium in the following study. SDF-1 level of liver lysates was 1.536 ± 0.339 ng/mL at 12 weeks. According to the results of SDF-1 induced MSCs migration* in vitro*, for neutralization studies the concentration of AMD3100 was chosen at 24 *μ*g/mL and anti-CXCR4 antibody at 20 *μ*g/mL. After adding AMD3100 (24 *μ*g/mL) or anti-CXCR4 antibody (20 *μ*g/mL), the number of migrated cells decreased to 13.8 ± 2.77 and 11.4 ± 2.70 (Figure 3(d)), however still higher than the number of migrated cells caused by liver lysates from untreated normal rats. These results demonstrated that SDF-1/CXCR4 axis involved in injured liver lysates induced BMSCs migration but not the only pathway.

### 3.5. SDF-1/CXCR4 Mediate BMSCs Migration in Rats

**Figure 4:**
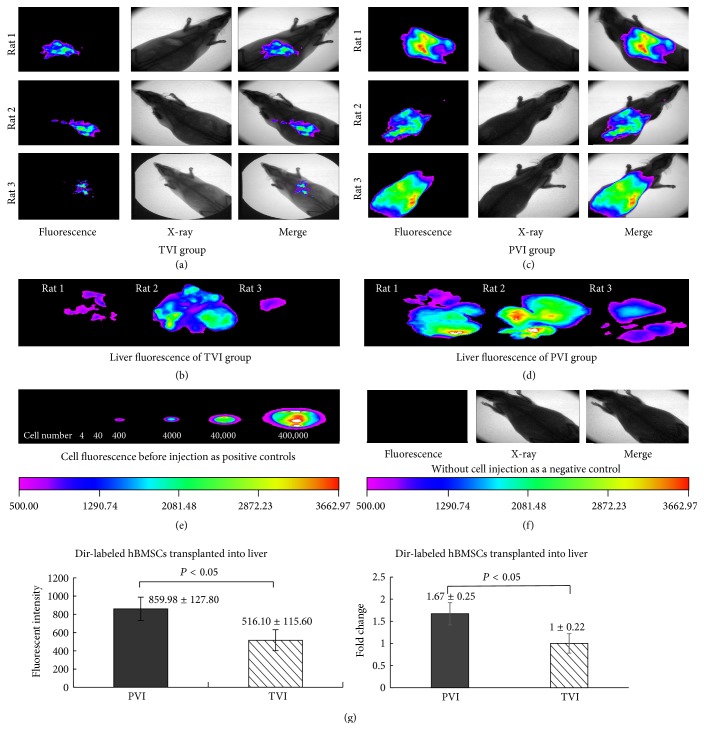
Difference between portal vein injection and tail vein injection. (a) The fluorescent intensity of whole rats; the BMSCs were injected through tail vein; (b) the fluorescent intensity of dissected livers; the BMSCs were injected through tail vein; (c) the fluorescent intensity of whole rats; the BMSCs were injected through portal vein; (d) the fluorescent intensity of dissected livers; the BMSCs were injected through portal vein; (e) Dir-labeled BMSCs before injection were as positive control; (f) rats without cell injection were as negative control; (g) the fluorescent intensity value and fold changes of the dissected livers of two groups; the difference was statistically significant.

We first compare the ways of cells injection. The cells were labeled with Dir dye, which gave out fluorescence in the liver tissues after cells injection and the fluorescence could be captured by* in vivo* imaging system. The fluorescent intensity of injured livers with cells injection through tail vein (TVI) was 516.10 ± 115.60 (Figures 4(a), 4(b), and 4(g)) and that through portal vein (PVI) was 859.98 ± 127.80 (Figures 4(c), 4(d), and 4(g)), which was significantly higher (fold change: 1.67 ± 0.25) (*P* < 0.05). Dir-labeled BMSCs before injection (Figure 4(e)) were as positive control and rats without cell injection were as negative control (Figure 4(f)). This result indicated that more cells migrated into liver through portal vein injection. So PVI injection was selected in the following transplantation study.

**Figure 5:**
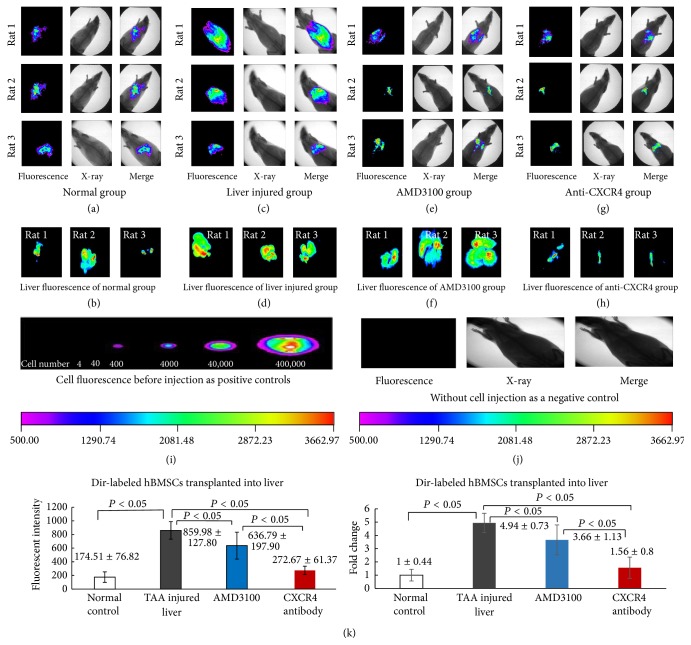
BMSCs migration in injured liver inhibited by anti-CXCR4 antibody and AMD3100. (a) The fluorescent intensity of normal rats after Dir-labeled BMSCs injection; (b) the fluorescent intensity of dissected normal livers after Dir-labeled BMSCs injection; (c) the fluorescent intensity of rats with TAA induced liver injury after Dir-labeled BMSCs injection; (d) the fluorescent intensity of dissected TAA injured livers after Dir-labeled BMSCs injection; (e) the fluorescent intensity of rats with TAA induced liver injury after Dir-labeled BMSCs and AMD3100 injection at the same time; (f) the fluorescent intensity of dissected injured livers after Dir-labeled BMSCs and AMD3100 injection at the same time; (g) the fluorescent intensity of rats with TAA induced liver injury after anti-CXCR4 antibody pretreated and Dir-labeled BMSCs injection; (h) the fluorescent intensity of dissected injured livers after anti-CXCR4 antibody pretreated and Dir-labeled BMSCs injection; (i) Dir-labeled BMSCs before injection were as positive control; (j) rats without cell injection were as negative control; (k) the fluorescent intensity value and fold changes of the dissected livers of each group; the differences were statistically significant.

We also compared the cell migration between normal liver and injured liver. After cells injection, the fluorescent intensity of normal livers (174.51 ± 76.82) (Figures 5(a), 5(b), and 5(k)) was statistically significantly lower than that of injured livers (859.98 ± 127.80) (compared with normal control, fold change: 4.94 ± 0.73) (Figures 5(c), 5(d), and 5(k)) (*P* < 0.05). More cells migrated into injured liver. The SDF-1 level of injured liver increased. So, the question is whether the migration was regulated by SDF-1/CXCR4 axis. First, CXCR4 antagonist AMD3100 was injected into liver through PV and then followed by BMSCs injection. The fluorescent intensity of the livers declined to 636.79 ± 197.90 (compared with normal control, fold change: 3.66 ± 1.13) (Figures 5(e), 5(f), and 5(k)), which indicated that AMD3100 significantly inhibited cells migration into the liver. And then the cells were incubated with anti-CXCR4 antibody to neutralize CXCR4 receptor before injection. The cell migration was also significantly inhibited by CXCR4 neutralization, the fluorescent intensity of the livers decreased to 272.67 ± 61.37 (compared with normal control, fold change: 1.56 ± 0.8) (*P* < 0.05) (Figures 5(g), 5(h), and 5(k)). Dir-labeled BMSCs before injection (Figure 5(i)) were as positive control and rats without cell injection were as negative control (Figure 5(j)). These findings suggested that SDF-1/CXCR4 plays a role in the migration of BMSCs into injured liver.

## 4. Discussion

Recently, studies have demonstrated the potential therapeutic function of BMSCs transplantation to treat acute and chronic liver injury. Because BMSCs produce new hepatocytes [18], secrete trophic factors to promote liver tissue repair [19], and modulate inflammatory responses [20]. BMSCs transplantation is a promising candidate for cell therapy in liver diseases. Cell homing and engraftment into the host liver are integral to cell-based therapies. The mechanism of BMSCs migration to injured liver is complicated and still not fully understood. In the current study, we try to investigate the role of SDF-1/CXCR4 axis in the recruitment of BMSCs migration into TAA induced injured liver. We revealed SDF-1 induced BMSC migration* in vitro* study. Our study found SDF-1 level gradually increased in rat model of TAA induced liver injury. And we demonstrated SDF-1/CXCR4 axis involved in BMSCs migration into the injured liver* in vitro* and* in vivo*.

CXCR4 is a G protein-linked seven-transmembrane spanning receptor, which is expressed on the surface of a small proportion of MSCs. Some studies indicated that most CXCR4 are intracellular storage, only small part of functional CXCR4 located on the cell surface [21, 22]. Our study was consistent with other studies, we found only small part of BMSCs expressed CXCR4 on the surface. After permeabilization, all the cells were CXCR4 positive which included intracellular and membrane CXCR4. It is believed that the receptor expression is gradually decreased as cells are expanded* in vitro* [23, 24]. In our study, CXCR4 was stably expressed from passages 1 to 8. And all the cells used in this study were below passage 8.

Several signal pathways have been implicated in the recruitment of BMSCs into injured organs; one of them is SDF-1/CXCR4 axis. After injury, SDF-1 increased in many tissues which recruit BMSCs to the site of injury [25–27]. And SDF-1/CXCR4 pathway is crucial in the migration of BMSCs into the injured organs. In* in vitro* study, we demonstrated that SDF-1 could induce BMSCs migration which was blocked by neutralizing anti-CXCR4 antibody or SDF-1 antagonist AMD3100. These results confirmed that SDF-1/CXCR4 involved BMSCs migration* in vitro*. We found SDF-1 levels of liver tissues increased after TAA induced injury. Other studies also indicated SDF-1 was increased in many tissues after injury [25–27]. BMSCs migration was observed by injury liver lysates* in vitro*. So the question raised whether the BMSCs migration was caused by upregulated SDF-1 in the liver injury. In order to answer this question, CXCR4 antagonist AMD3100 or anti-CXCR4 antibody was added in the medium of liver lysates to neutralize the binding site of SDF-1. After AMD3100 or anti-CXCR4 antibody was added, the cells migration induced by the liver lysates was partially inhibited. In* in vivo* study, after BMSCs were incubated with anti-CXCR4 antibody before injection, or AMD3100 was injected at the same time during BMSCs injection, the migration of BMSCs towards injured liver was also partially blocked. These results demonstrated that SDF-1 of liver injury and SDF-1/CXCR4 axis involved in BMSCs migration to injured liver; however, it is not the only pathway. Other studies indicated proteolytic enzymes MMP-2 and MMP-9 and cytokines HGF are also involved in BMSCs migration [28–30].

The conventional methods of tracking transplanted cells require histological immune-staining or real-time PCR for gene detection* in vitro*, which are time consuming and of low efficiency. The* in vivo* imaging system is a relatively new optical imaging system that is being widely used for cell tracking, allowing noninvasive cell detection and cell migration monitoring [31, 32]. There is also no necessity for detecting cells by immunohistochemistry in thousands of slides. This is whole-body imaging technique for monitoring transplanted cells. In our preliminary experiment, we found that the fluorescent intensity of the liver gradually increased and reached peak at 24 h. So, in our study, the fluorescent intensity of the rats was checked at 24 h after cell injection. Not only the fluorescence intensity of whole rats was checked, but the dissected livers as well, which were perfused with saline to remove contaminating blood which might contain unmigrated BMSCs. The results of whole body experiments and dissected livers were consistent. And both of them demonstrated that SDF-1/CXCR4 axis was involved in BMSCs migration into the injured liver.

The studies we report here demonstrate that the level of SDF-1 increased in liver injury and SDF-1/CXCR4 axis is involved in BMSCs migration toward the injured liver.

## Abbreviation

BMSCs: Bone marrow mesenchymal stromal stem cells
SDF-1: Stromal cell-derived factor-1
HGF: Hepatocellular growth factor
SD rats: Sprague-Dawley rats
AEEC: Animal Experimentation Ethics Committee
TAA: Thioacetamide
DiR: 1,1-Dioctadecyl-3,3,3,3-tetramethylindotricarbocyanine iodide
ELISA: Enzyme-linked immunosorbent assay
PVI: Portal vein injection
TVI: Tail vein injection.
